# Pomolic Acid from the Dong Botanical Drug Madeng’ai Suppresses TNF-α-Induced Inflammatory Response in RA-HFLS by Inhibiting NF-κB Signaling Pathway Activation

**DOI:** 10.3390/molecules31101560

**Published:** 2026-05-08

**Authors:** Sisi Huang, Wei Cai, Yan Wang, Xiaoliang Xing, Zaiqi Zhang

**Affiliations:** 1 College of Life Sciences, Jishou University, Jishou 416000, China; H2403312922@163.com (S.H.); 17774370317@163.com (Y.W.); 2 Hunan Provincial Key Laboratory of Dong Medicine, Hunan University of Medicine, Huaihua 418000, China; 20120941161@bucm.edu.cn

**Keywords:** pomolic acid, rheumatoid arthritis, fibroblast-like synoviocytes, NF-κB signaling pathway, inflammatory cytokines

## Abstract

**Objective:** This study aimed to investigate whether pomolic acid (PA), a predicted bioactive metabolite of the Dong botanical drug Madeng’ai (MDA), suppresses inflammatory cytokine expression by inhibiting nuclear factor-κB (NF-κB) pathway activation in a tumor necrosis factor-α (TNF-α)-induced human rheumatoid arthritis fibroblast-like synoviocyte (RA-HFLS) model. **Methods:** PA content in MDA from different regions and harvest years was quantified via High-Performance Liquid Chromatography (HPLC). Network analysis was employed as a hypothesis-generating tool to predict potential targets and pathways, followed by molecular docking to validate the binding affinity of PA to core targets of the NF-κB pathway, and ADMET prediction to evaluate its pharmacokinetic properties and safety profile. The RA-HFLS inflammatory model was induced by TNF-α. Cell viability and inflammatory cytokine secretion were assessed using Cell Counting Kit-8 (CCK-8) and enzyme-linked immunosorbent assay (ELISA). NF-κB signaling pathway activation and downstream gene expression were examined by Western blot and reverse transcription quantitative polymerase chain reaction (RT-qPCR), respectively. **Results:** HPLC analysis revealed that MDA samples from Guizhou harvested in 2019 contained the highest PA content (0.1117 mg/g). Network analysis predicted the NF-κB signaling pathway as a candidate mechanism underlying PA’s potential anti-inflammatory effects. Molecular docking showed that PA stably bound to IKKβ, p65, and IκBα, while ADMET prediction indicated favorable intestinal absorption, low drug–drug interaction risk, and good genetic safety, albeit with potential hepatotoxicity and reproductive toxicity risks. In the TNF-α-induced RA-HFLS model, PA dose-dependently inhibited abnormal cell proliferation and significantly reduced the secretion of pro-inflammatory cytokines TNF-α and interleukin-6 (IL-6). Mechanistic studies indicated that PA suppressed the activation of the NF-κB signaling pathway, thereby downregulating the mRNA expression of inflammatory genes such as IL-6, TNF-α, and interleukin-1β (IL-1β). **Conclusions:** PA, a bioactive metabolite of the Dong botanical drug MDA, may inhibit NF-κB signaling pathway activation, thereby downregulating TNF-α-induced inflammatory cytokine expression in RA-HFLS, demonstrating its in vitro anti-inflammatory potential.

## 1. Introduction

Rheumatoid arthritis (RA) is a chronic systemic autoimmune disease characterized by persistent synovitis, progressive joint destruction, and systemic complications, with an estimated global prevalence of approximately 1% [1]. It not only severely impairs patients’ joint function and quality of life but also imposes a significant economic burden on healthcare systems worldwide. The pathogenesis of RA is highly complex, involving interplay among immune cell imbalance, inflammatory cytokine network dysregulation, and aberrant activation of signaling pathways [2]. Meanwhile, a cascade network of pro-inflammatory cytokines, including tumor necrosis factor-α (TNF-α), interleukin-1β (IL-1β), and interleukin-6 (IL-6), further amplifies local inflammation and propels disease progression [3].

Previous studies indicate that abnormal activation of various signaling pathways, including nuclear factor-κB (NF-κB), mitogen-activated protein kinase (MAPK), and Janus kinase-signal transducer and activator of transcription (JAK-STAT) pathways, represents a central feature in RA pathogenesis [4]. The NF-κB signaling pathway, for example, is one of the master regulators of inflammatory responses: upon activation by pro-inflammatory stimuli such as TNF-α, it translocates to the nucleus and promotes the transcription of multiple target genes, including those encoding TNF-α, IL-6, and IL-1β, thereby creating a positive feedback loop that amplifies local inflammation and drives disease progression [5]. Such aberrant activation of these pathways may promote the expression of pro-inflammatory cytokines and matrix metalloproteinases, mediate immune cell activation and cytokine secretion, and exacerbate synovial inflammation and extracellular matrix degradation [4]. Consequently, these pathways have become important therapeutic targets for RA. Current clinical RA treatments include non-steroidal anti-inflammatory drugs, disease-modifying antirheumatic drugs, and biologics. However, these treatments have limitations, such as insufficient efficacy, adverse effects, and high costs [6]. These drawbacks underscore the need for continued exploration of natural bioactive metabolites with diverse sources, favorable safety profiles, and the ability to multi-target the RA pathological network.

Natural metabolites hold promise for autoimmune disease treatment due to their unique advantages of multi-target regulation, low toxicity, and structural diversity [7]. Botanical drugs, as an integral part of traditional Chinese medicine, represent a valuable repository of active natural metabolites. Madeng’ai (MDA, a variant of *Potentilla freyniana* Bornm.), a botanical drug commonly used by the Dong ethnic group in Guizhou and Hunan provinces, has demonstrated anti-inflammatory and analgesic properties in folk practice, with preliminary pharmacological validation in modern studies [8]. Among its constituent metabolites, pomolic acid (PA), a pentacyclic triterpenoid, has been identified as a potential bioactive metabolite. Previous phytochemical studies have demonstrated that PA exhibits moderate anti-proliferative activity against human rheumatoid arthritis fibroblast-like synoviocyte (RA-HFLS), with a reported half-maximal inhibitory concentration (IC50) of 25.12 ± 1.97 μM [8]. In addition, pomolic acid exhibits quantifiable anti-inflammatory effects in vivo. Schinella et al. demonstrated that PA reduces carrageenan-induced paw edema in mice and inhibits in vivo IL-1β production by 39% [9]. Furthermore, Banno et al. reported that it inhibits 12-O-tetradecanoylphorbol-13-acetate-induced ear inflammation in mice, with a 50% inhibitory dose (ID50) of 0.12 mg/ear [10]. However, whether PA exerts similar anti-inflammatory effects in RA-HFLS and the mechanisms involved remain unknown. To address this gap, the present study employed an integrated approach combining network analysis, molecular docking, and absorption, distribution, metabolism, excretion, and toxicity (ADMET) prediction, followed by in vitro validation in a cellular model of RA inflammation. The aim was to systematically investigate the inhibitory effects of PA on TNF-α-induced inflammatory responses in RA-HFLS and elucidate the associated molecular mechanisms.

## 2. Results

### 2.1. Determination of PA Content in MDA

**Figure 1:**
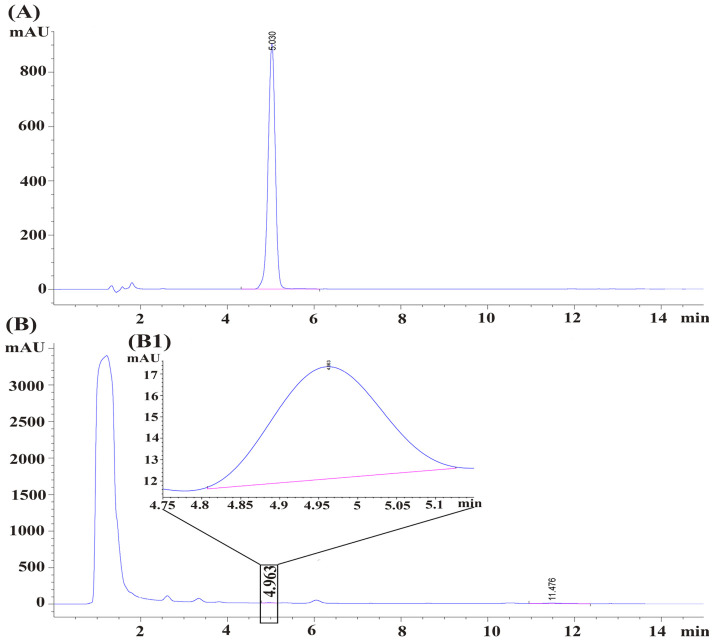
**The chromatograms of pomolic acid (PA) and Madeng’ai (MDA).** (**A**) Chromatogram of the PA standard; (**B**) Full-range chromatogram of MDA sample; (**B1**) Enlarged view of the PA peak region in (**B**).

The established High-Performance Liquid Chromatography (HPLC) method was used for quantitative analysis of PA in different MDA batches. Representative chromatograms (Figure 1) showed stable retention times, symmetrical peaks, and good separation from adjacent impurities, indicating strong specificity for accurate PA quantification. Method validation demonstrated excellent linearity for PA within the concentration range of 0.00414 to 2.12 mg/mL. The standard curve equation was Y = 4841.1X + 30.322 (R^2^ = 0.9999), confirming a reliable linear relationship.

Quantitative analysis of three MDA batches with different geographical origins and harvest years (Table 1) indicated that the accumulation of PA was significantly influenced by environmental factors. The sample collected from Youzha Street, Guizhou, in 2019 (batch CL-17-1-1) exhibited the highest average PA content (0.1117 mg/g), followed by the sample from Tongdao County, Huaihua, Hunan, harvested in 2023 (batch CL-17-1-7). In contrast, the lowest PA content was found in the sample from the same location harvested in 2024 (batch CL-17-1-8). These results suggest that both the geographical origin and the harvest year are critical variables affecting the PA content in MDA. Furthermore, this study identified a batch with higher PA content for subsequent experimental investigations.

### 2.2. Network Analysis Prediction of Potential Anti-RA Targets and Mechanisms of PA

**Figure 2:**
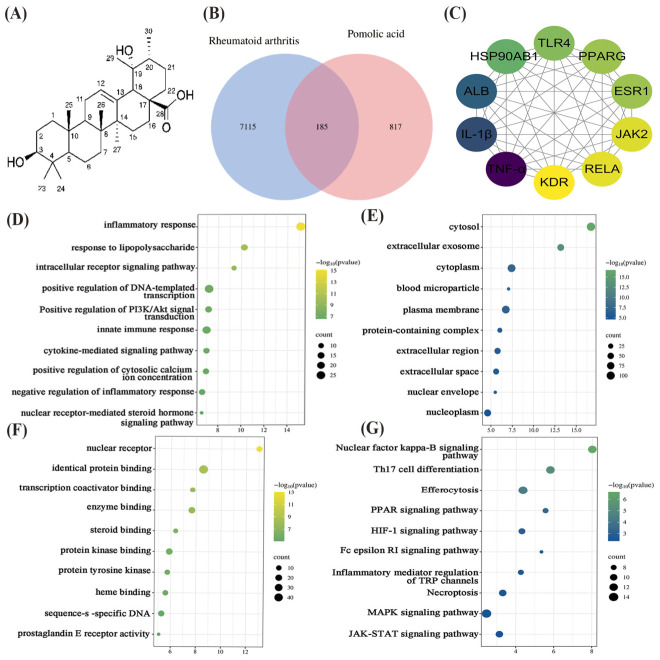
**Network analysis prediction of potential anti-RA targets and mechanisms of PA.** (**A**) Chemical structure of PA. (**B**) Venn diagram illustrating the intersection between predicted targets of PA (from the Traditional Chinese Medicine Systems Pharmacology (TCMSP) database and SwissTargetPrediction) and known rheumatoid arthritis (RA)-related targets (from GeneCards and Online Mendelian Inheritance in Man (OMIM) databases). (**C**) Protein-protein interaction (PPI) network of the 185 intersecting targets. Node color intensity corresponds to the degree value (connectivity), with darker colors indicating higher connectivity. (**D**–**F**) Gene Ontology (GO) enrichment analysis of the intersecting targets: (**D**) Biological Processes, (**E**) Cellular Components, (**F**) Molecular Functions. Bars represent −log10 (*p* value), with higher values indicating greater enrichment significance. (**G**) Kyoto Encyclopedia of Genes and Genomes (KEGG) pathway enrichment analysis of the intersecting targets. Bubble size represents the number of genes enriched in each pathway, and color represents −log10 (*p* value).

To identify potential molecular targets and mechanisms of PA against RA, we performed a network analysis integrating metabolite target prediction and disease gene databases. It is important to note that all predicted targets require experimental validation. A total of 1002 potential PA targets were predicted via the Traditional Chinese Medicine Systems Pharmacology (TCMSP) and SwissTargetPrediction. From the GeneCards and Online Mendelian Inheritance in Man (OMIM) databases, 7300 RA-related disease targets were retrieved using “rheumatoid arthritis” as the keyword, with a relevance score ≥ 5 applied for GeneCards data. Venn diagram analysis identified 185 common targets (Figure 2B) as potential targets of PA against RA. To visualize the interactions among these potential targets, a protein-protein interaction (PPI) network was constructed by importing the 185 intersecting targets into the Search Tool for the Retrieval of Interacting Genes/Proteins (STRING) database. As shown in the PPI network (Figure 2C), RELA (also known as NF-κB p65), Toll-like receptor 4 (TLR4), and Janus kinase 2 (JAK2) were identified as potential core targets, each directly interacting with pro-inflammatory cytokines such as TNF-α and IL-1β. Based on these interactions and established biological knowledge, it is suggested that PA may exert its anti-inflammatory effects in RA-related cellular contexts by modulating core signaling proteins (RELA, TLR4, JAK2), which in turn regulate the expression of downstream inflammatory effectors like TNF-α and IL-1β.

To further explore the potential biological processes and signaling pathways involved, we performed Gene Ontology (GO) enrichment analysis and Kyoto Encyclopedia of Genes and Genomes (KEGG) pathway enrichment analysis on the 185 intersecting targets using the Database for Annotation, Visualization and Integrated Discovery (DAVID) database. Enrichment significance was set at a false discovery rate (FDR)-corrected *p* < 0.05. GO enrichment analysis identified a set of significantly enriched terms across three categories (Figure 2D–F). The enriched biological processes primarily included inflammatory response, response to lipopolysaccharide, cytokine-mediated signaling pathway, and innate immune response. Enriched cellular components mainly comprised the cytosol, plasma membrane, extracellular space, and nucleoplasm. Enriched molecular functions were predominantly nuclear receptor activity, identical protein binding, protein kinase binding, and sequence-specific DNA binding. These terms are highly relevant to the pathological processes of inflammatory activation and immune regulation in RA. KEGG pathway enrichment analysis revealed several significantly enriched signaling pathways (Figure 2G). Ranked by −log10 (*p* value), the NF-κB signaling pathway exhibited the highest enrichment significance. This was followed by Th17 cell differentiation, efferocytosis, the PPAR signaling pathway, and the HIF-1 signaling pathway. The MAPK and JAK-STAT signaling pathways were also significantly enriched. Among these, NF-κB, MAPK, and JAK-STAT are classic pathways involved in the regulation of RA inflammation, while Th17 cell differentiation is closely related to RA immune imbalance.

Collectively, these results suggest that PA may target a multi-pathway synergistic network, with the NF-κB pathway as the most significantly enriched candidate mechanism. These predictions provide testable hypotheses that require experimental validation in cellular systems.

### 2.3. Molecular Docking and ADMET Prediction Results

**Figure 3:**
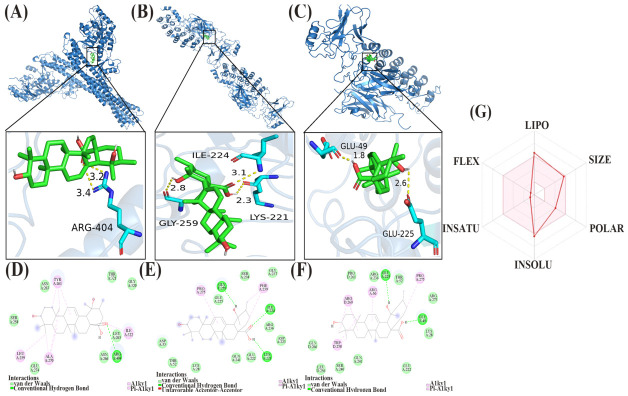
Molecular docking results of PA with core targets of the inhibiting nuclear factor-κB (NF-κB) pathway and bioavailability radar analysis. (**A**–**C**) Three-dimensional binding mode diagrams of pomolic acid with inhibiting nuclear factor-κB (NF-κB), p65 (RELA, NF-κB subunit), and inhibitor of κB α (IκBα). PA is shown as green sticks, the proteins are represented as blue ribbons, and hydrogen bonds are indicated by yellow dashed lines. (**D**–**F**) Two-dimensional interaction diagrams of PA with IKKβ, p65, and IκBα. Hydrogen bonds are indicated by green dashed lines, and hydrophobic interactions are indicated by pink dashed lines. (**G**) Bioavailability radar of PA generated by SwissADME. The pink area represents the ideal range for oral drug parameters (LIPO: lipophilicity, SIZE: molecular weight, POLAR: polarity, INSOLU: water solubility, INSATU: unsaturation, FLEX: flexibility). The red line indicates the measured values of PA; falling completely within the pink area indicates favorable drug-likeness.

Based on the network pharmacology predictions, inhibitor of κB kinase β (IKKβ) (PDB: 4KIK), p65 (PDB: 1NFI), and inhibitor of κB α (IκBα) (PDB: 1IKN, p65/IκBα complex) were selected as receptor proteins, with PA as the ligand for molecular docking analysis. The docking results showed that the binding energies of PA to IKKβ, p65, and IκBα were −8.8, −7.8, and −7.9 kcal/mol, respectively, all below −5.0 kcal/mol, indicating stable binding between PA and the three targets (Table 2). Two-dimensional interaction analysis (Figure 3D–F) revealed multiple non-covalent interactions, including hydrogen bonds, hydrophobic interactions, and van der Waals forces, between PA and each target. Three-dimensional binding mode analysis (Figure 3A–C) showed that PA embedded into the active pocket regions of each protein: it bound near the ATP-binding pocket of IKKβ, the DNA recognition and transcriptional activation region of p65, and the p65-IκBα interaction interface of IκBα. Collectively, the molecular docking results confirmed stable binding of PA to core proteins of the NF-κB pathway at the structural level, providing a molecular basis for its inhibitory effects on IκBα phosphorylation and p65 activation observed in vitro.

ADMET prediction was performed using three online platforms: SwissADME, admetSAR 3.0, and ProTox-3.0 (Table 3). The molecular weight and topological polar surface area (TPSA) of PA met the requirements for oral drugs, with high lipophilicity (LogP = 5.15) but poor water solubility. The bioavailability radar (Figure 3G) indicated favorable theoretical bioavailability for PA, falling within the ideal range for oral drugs. The compound exhibited high gastrointestinal absorption, did not cross the blood–brain barrier, and showed low risk of central nervous system toxicity. It was identified as a P-glycoprotein substrate but did not inhibit major CYP450 enzymes, suggesting a low risk of drug–drug interactions. Drug-likeness analysis indicated that PA violated one Lipinski rule, complied with Veber rules, had a bioavailability score of 0.56, and contained no Pan-Assay Interference Compounds (PAINS) alerts, indicating favorable drug-likeness. Toxicity predictions showed negative results for Ames mutagenicity (Ames) mutagenicity, skin sensitization, and hERG cardiotoxicity at low concentrations, indicating good genetic safety; however, potential risks, including acute oral toxicity, hepatotoxicity, respiratory toxicity, reproductive toxicity, and repeated-dose toxicity, were noted, warranting careful dose control and further experimental validation. Collectively, the ADMET predictions suggest that PA possesses favorable intestinal absorption, low risk of drug–drug interactions, and good genetic safety, supporting its potential as a lead compound for further development.

### 2.4. In Vitro Validation of the Anti-Inflammatory Effects of PA

**Figure 4:**
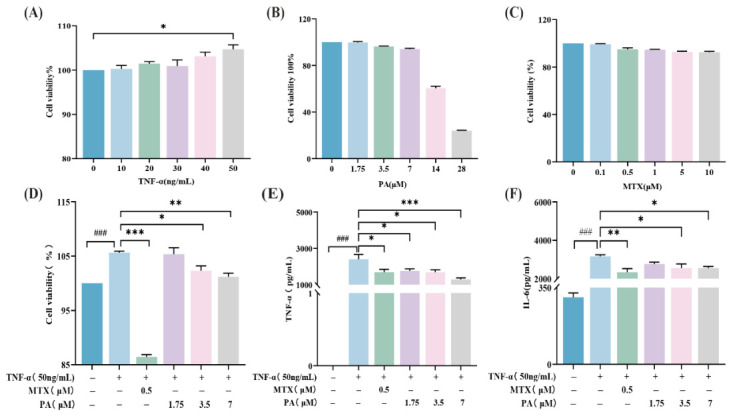
**Effects of PA on the viability, proliferation, and inflammatory cytokine secretion in human rheumatoid arthritis fibroblast-like synoviocyte (RA-HFLS) cells.** (**A**) Effect of different concentrations of tumor necrosis factor-α (TNF-α) on RA-HFLS cell viability after 24 h stimulation; (**B**,**C**) Cytotoxicity assessment of RA-HFLS cells treated with different concentrations of PA or Methotrexate (MTX) for 24 h; (**D**) Inhibitory effects of PA and MTX on TNF-α-induced proliferation of RA-HFLS cells; (**E**,**F**) Concentrations of TNF-α and interleukin-6 (IL-6) proteins in cell culture supernatant measured by enzyme-linked immunosorbent assay (ELISA) after 24 h treatment. Data are presented as the mean ± SEM (*n* = 3–4). ### *p* < 0.001 versus the Control group; * *p* < 0.05, ** *p* < 0.01, *** *p* < 0.001 versus the TNF-α Model group.

The Cell Counting Kit-8 (CCK-8) assay was performed to evaluate the effect of different concentrations of TNF-α on RA-HFLS viability. The results (Figure 4A) indicated no significant changes in cell viability at concentrations of 10, 20, 30, or 40 ng/mL TNF-α compared to the Control group. In contrast, treatment with 50 ng/mL TNF-α significantly increased cell viability, establishing this concentration as optimal for inducing proliferation.

PA Cytotoxicity and IC50 determination: Compared to the Control group, cell viability remained above 90% after treatment with 1.75, 3.5, and 7 µM PA (99.74% ± 0.79%, 96.28% ± 0.44%, and 94.03% ± 0.79%, respectively) (Figure 4B). Higher concentrations (14 and 28 µM PA) caused a marked decrease in cell viability. Based on the dose–response curve across the concentration range of 1.75–28 μM, the IC50 value of PA in RA-HFLS cells was calculated to be 17.18 μM (95% confidence interval (CI): 16.43–17.98 μM) using nonlinear regression analysis. This value is consistent with the previously reported IC50 of 25.12 ± 1.97 μM in RA-FLS cells, with the minor difference likely attributable to variations in cell passage number, culture conditions, or assay protocols. Therefore, 1.75, 3.5, and 7 µM were selected as the low (PA-L), medium (PA-M), and high (PA-H) concentrations for subsequent experiments. MTX Cytotoxicity: Compared to the Control group, cell viability remained above 90% across the concentration range of 0.1 to 10 µM Methotrexate (MTX) (Figure 4C). Treatment with 0.1 µM MTX resulted in 99.25% ± 0.49% cell viability. However, this concentration did not show a significant inhibitory effect in the TNF-α-induced RA-HFLS proliferation model. Therefore, 0.5 µM MTX, which maintained 95% ± 1.22% viability, was selected as the effective positive control concentration for subsequent comparative experiments.

Effect on TNF-α-induced Proliferation: The CCK-8 assay results demonstrated the effect of PA on TNF-α-induced RA-HFLS cell proliferation (Figure 4D). Compared to the Control group, the Model group (treated with 50 ng/mL TNF-α) exhibited a significant increase in cell proliferation viability, with statistical significance, confirming the successful establishment of the inflammatory model. Compared to the Model group, the MTX group showed the strongest inhibitory effect, with a statistically significant reduction in proliferation viability. The PA-L group (1.75 µM) showed no significant change. In contrast, both the PA-M (3.5 µM) and PA-H (7 µM) groups demonstrated a significant and concentration-dependent inhibition of proliferation compared to the Model group, with statistical significance. These results indicate that PA exerts a significant inhibitory effect on TNF-α-induced RA-HFLS cell proliferation, and the inhibitory effect of the positive control drug MTX was stronger.

ELISA for Cytokine Secretion: The effects of PA on TNF-α and IL-6 secretion by RA-HFLS cells were assessed by enzyme-linked immunosorbent assay (ELISA) (Figure 4E,F). Compared to the Control group, the Model group exhibited significantly elevated levels of both TNF-α and IL-6 in the culture supernatant, confirming that TNF-α stimulation successfully induced inflammatory cytokine secretion. Following PA treatment, the secretion of TNF-α decreased in a clear concentration-dependent manner. Statistically significant differences were observed for the MTX group and all PA-treated groups (PA-L, PA-M, and PA-H) compared to the Model group (Figure 4E). Since the same concentration of exogenous TNF-α (50 ng/mL) was present in all groups, the differences in measured TNF-α levels among groups reflect changes in endogenous TNF-α secretion by RA-HFLS cells. For IL-6, its secretion also displayed a concentration-dependent decrease with increasing PA concentrations. Compared to the Model group, statistically significant reductions were observed in the MTX, PA-M, and PA-H groups, while no significant difference was found in the PA-L group (Figure 4F).

### 2.5. In Vitro Validation of the Anti-Inflammatory Mechanism of PA

**Figure 5:**
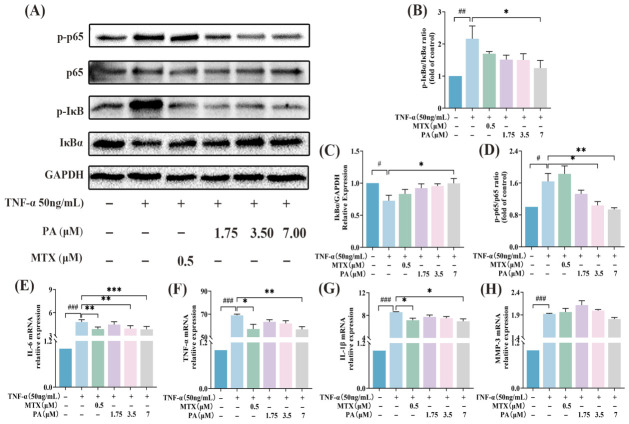
Effects of PA on key proteins in the NF-κB signaling pathway and on the mRNA expression of inflammatory cytokines. (**A**) Representative Western blot (WB) images showing p-p65 (65 kDa), p65 (65 kDa), p-IκBα (39 kDa), IκBα (39 kDa), and GAPDH (37 kDa). (**B**) Quantitative analysis of p-IκBα/IκBα ratio, reflecting IKK-mediated phosphorylation normalized to remaining IκBα; (**C**) Quantitative analysis of IκBα/glyceraldehyde-3-phosphate dehydrogenase (GAPDH) ratio, reflecting IκBα degradation; (**D**) Quantitative analysis of p-p65/p65 ratio, reflecting p65 activation. (**E**–**H**) Relative mRNA expression levels of IL-6, interleukin-1β (IL-1β), TNF-α, and matrix metalloproteinase-3 (MMP-3) in RA-HFLS cells detected by reverse transcription quantitative polymerase chain reaction (RT-qPCR). Data are presented as mean ± SEM (*n* = 4). # *p* < 0.05, ## *p* < 0.01, ### *p* < 0.001 versus the Control group; * *p* < 0.05, ** *p* < 0.01, *** *p* < 0.001 versus the TNF-α Model group.

Western blot (WB) analysis was performed to evaluate the effects of PA on the expression of key NF-κB pathway proteins, including p-p65, p65, p-IκBα, and IκBα, in RA-HFLS cells. The results (Figure 5B–D) confirmed that, compared with the Control group, the Model group exhibited a significantly elevated p-IκBα/IκBα ratio, a concomitant significant decrease in the IκBα/glyceraldehyde-3-phosphate dehydrogenase (GAPDH) ratio, and a marked increase in the p-p65/p65 ratio (all with statistical significance). This verified that TNF-α successfully activated the NF-κB signaling pathway by inducing IκBα phosphorylation, IκBα degradation, and p65 phosphorylation, thereby triggering downstream inflammatory responses. For the p-IκBα/IκBα ratio (Figure 5B), only the PA-H group displayed a significant reduction relative to the Model group, whereas the PA-L, PA-M, and MTX groups showed no significant changes. This suggests that high-concentration PA specifically inhibits the initial step of TNF-α-induced IκBα phosphorylation. Subsequently, regarding the IκBα/GAPDH ratio (Figure 5C), the PA-H group showed a significant increase compared with the Model group, while the MTX, PA-L, and PA-M groups only exhibited an upward trend without statistical significance. This indicates that, as a consequence of inhibiting phosphorylation, high-concentration PA effectively reverses TNF-α-induced IκBα degradation. Finally, for the p-p65/p65 ratio (Figure 5D), both the PA-M and PA-H groups exhibited significant, concentration-dependent decreases compared with the Model group, while the PA-L group showed no significant change. Notably, the MTX group presented an increased p-p65/p65 ratio relative to the Model group, which may imply concentration- or cell type-specific regulatory effects of MTX on the NF-κB pathway. Collectively, these results demonstrate that PA blocks NF-κB signaling pathway activation by inhibiting the upstream IκBα phosphorylation and its subsequent degradation, as well as the downstream p65 phosphorylation, thereby suppressing the transcriptional expression of downstream inflammatory genes and exerting anti-inflammatory effects.

Reverse transcription quantitative polymerase chain reaction (RT-qPCR) was performed to assess the effects of PA on the mRNA expression levels of IL-6, TNF-α, IL-1β, and MMP-3 in RA-HFLS cells. The results (Figure 5E–H) showed that, compared to the Control group, the Model group exhibited significantly increased mRNA expression levels of IL-6, TNF-α, IL-1β, and lMatrix Metalloproteinase-3 (MMP-3), all with statistical significance. This indicates that TNF-α successfully induced the transcriptional activation of inflammatory cytokines and matrix metalloproteinase genes, further validating the successful establishment of the inflammatory model. IL-6 mRNA Expression: Compared to the Model group, IL-6 mRNA expression was significantly reduced in the MTX, PA-M, and PA-H groups, with statistical significance. The PA-L group showed no significant change. The inhibitory effect strengthened as PA concentration increased, indicating a concentration-dependent regulatory trend (Figure 5E). TNF-α mRNA Expression: Compared to the Model group, TNF-α mRNA expression was significantly reduced in the MTX and PA-H groups, with statistical significance. No significant difference was observed between the PA-L and PA-M groups, but a concentration-dependent decreasing trend was noted (Figure 5F). IL-1β mRNA Expression: Compared to the Model group, IL-1β mRNA expression was significantly reduced in the MTX and PA-H groups, with statistical significance. The PA-L and PA-M groups individually showed no significant change, but collectively exhibited a concentration-dependent inhibitory trend (Figure 5G). MMP-3 mRNA Expression: A statistically significant difference in MMP-3 mRNA expression was observed only between the Model and Control groups. No significant differences were found between any drug treatment group (MTX, PA-L, PA-M, PA-H) and the Model group. Notably, MMP-3 mRNA levels in the PA-L and PA-M groups were slightly elevated compared to the Model group, while the PA-H group showed a slight inhibitory trend (Figure 5H). This suggests that PA has a relatively weak regulatory effect on MMP-3 gene expression, potentially exhibiting a biphasic regulatory pattern characterized by low-concentration promotion and high-concentration inhibition. The specific underlying mechanism requires further investigation.

## 3. Discussion

Persistent excessive inflammation in synovial tissue is a core pathological feature of RA. The cascade network of pro-inflammatory cytokines such as TNF-α, IL-6, and IL-1β in the RA synovium not only directly causes tissue damage but also amplifies inflammation via positive feedback loops, driving disease progression [3]. Therefore, targeting this key inflammatory network is a central strategy in anti-RA therapy. This study found that PA, a bioactive metabolite of MDA, can effectively intervene in this inflammatory network.

To establish the RA-HFLS inflammatory model, we first screened TNF-α concentrations from 10 to 50 ng/mL. Under our experimental conditions, 50 ng/mL TNF-α significantly promoted cell proliferation, whereas concentrations of 10–40 ng/mL showed no significant effect. This concentration was selected for several reasons. First, 50 ng/mL TNF-α consistently induced robust proliferative responses and inflammatory cytokine expression in our system, ensuring model reliability. Second, although lower TNF-α concentrations (e.g., 1–10 ng/mL) have been reported to induce inflammatory responses in some RA-FLS studies [11], cellular sensitivity to TNF-α varies considerably depending on cell origin, passage number, and culture conditions. Third, local TNF-α levels in the synovial fluid of active RA patients can be substantially higher than systemic levels. Thus, a relatively high stimulation concentration, such as 50 ng/mL, more closely mimics the inflammatory microenvironment of the RA synovium [12]. Taken together, these observations indicate that 50 ng/mL TNF-α is appropriate for establishing the RA-HFLS inflammatory model under our experimental conditions. To ensure that the anti-inflammatory effects of PA were not due to basal cytotoxicity, we screened and confirmed three non-cytotoxic working concentrations of PA (1.75, 3.5, and 7 μM; cell viability > 90%) based on cell viability criteria [13]. Regarding the positive control, although 0.1–10 μM MTX showed no obvious cytotoxicity, the low concentration (0.1 μM) exhibited limited inhibitory effect in this model, consistent with literature reports [14]. Therefore, we ultimately selected 0.5 μM MTX as the positive control.

Under the aforementioned experimental models and conditions, this study demonstrated that PA concentration-dependently inhibited TNF-α-induced abnormal proliferation of RA-HFLS cells. ELISA results revealed that PA significantly reduced the protein secretion levels of IL-6 and TNF-α, while RT-qPCR further confirmed that PA downregulated the mRNA expression of IL-6, TNF-α, and IL-1β. The consistency between these findings collectively verifies the anti-inflammatory activity of PA in the RA synovial cell model. This observation holds significant implications. PA is widely distributed in plants of the *Potentilla* genus and represents one of the key bioactive metabolites responsible for their anti-inflammatory effects [15,16]. As a representative metabolite of this genus, PA is present at relatively high levels in MDA [8] and has been reported to exhibit broad anti-inflammatory, anti-tumor, and immunomodulatory activities [9]. For instance, it can alleviate acute inflammatory edema [9]. Notably, to our knowledge, this is the first study to systematically validate the anti-inflammatory effects of PA in an RA synovial cell model. This not only provides modern scientific support for the traditional anti-inflammatory applications of MDA but also suggests PA’s potential to intervene in RA-related inflammatory processes. Furthermore, these findings offer preliminary in vitro experimental evidence supporting the further investigation of MDA-derived PA as a potential anti-inflammatory agent for RA-related applications.

In the present study, network pharmacology predicted three potential core targets of PA against RA: RELA (NF-κB p65), TLR4, and JAK2. Among these, the NF-κB pathway was selected for experimental validation for the following reasons. First, KEGG enrichment analysis identified the NF-κB pathway as the most significantly enriched pathway among all predicted targets (Figure 2G), suggesting it is the most likely candidate mechanism. Second, while TLR4 and JAK2 are also recognized as important regulators of RA pathogenesis, they represent distinct signaling axes that require separate experimental designs. Validating multiple pathways is beyond the scope of the present study, which focuses on establishing the anti-inflammatory effect of PA in RA-HFLS cells. Third, the multiple pharmacological effects of PA, including its anti-inflammatory and anti-tumor activities, are closely associated with the regulation of several key signaling pathways, with the NF-κB pathway being a common target. In cancer research, PA has been shown to exert inhibitory effects through diverse mechanisms. For example, PA inhibits the migration and invasion of breast cancer cells by suppressing the NF-κB/ERK/mTOR pathways [17]. Beyond breast cancer, PA induces ferroptosis in non-small cell lung cancer [18], promotes apoptosis through autophagy in colon cancer while suppressing STAT3 [19], and overcomes drug resistance in prostate cancer by bypassing P-gp-mediated multidrug resistance [20]. In inflammatory diseases, PA similarly demonstrates multi-pathway regulatory capabilities. Specifically, PA promotes endotoxin tolerance in macrophages by upregulating IRG1 [21], alleviates acute lung injury through NF-κB inhibition [22], reduces renal fibrosis by targeting SMAD-STAT signaling [23]. Collectively, these findings suggest that the NF-κB signaling pathway is likely a common and key target mediating the pharmacological actions of PA across different pathological models. Therefore, the NF-κB pathway was prioritized in the present study, and the roles of TLR4 and JAK2 remain important directions for future investigation.

Building on this evidence, we investigated whether PA exerts its anti-inflammatory effects in RA through the NF-κB signaling pathway. The results demonstrated, for the first time in an RA-HFLS model, that PA inhibits NF-κB signaling pathway activation by suppressing IκBα phosphorylation and degradation, as well as subsequent p65 phosphorylation. It is noteworthy that the p-p65/p65 ratio in the positive control MTX-treated group was actually higher than that in the Model group, a phenomenon consistent with reported cell-specific differences in MTX’s regulation of the NF-κB signaling pathway [24]. Studies indicate that in RA-HFLS, MTX may primarily inhibit NF-κB activity indirectly via adenosine receptor signaling, rather than by directly reducing p-p65 protein expression [25]. This contrast further highlights the mechanistic differences between PA and the classic drug MTX. The excessive production of inflammatory cytokines in RA synovial cells is closely related to the central event of abnormal, persistent activation of the NF-κB signaling pathway [26]. Therefore, PA’s direct inhibition of the NF-κB pathway forms the molecular basis for its effective downregulation of downstream inflammatory cytokines such as IL-6, TNF-α, and IL-1β. This mechanistic alignment with established anti-RA natural metabolites, such as paeonol [27] and diosmetin [28], not only provides crucial in vitro molecular pharmacological support for the further exploration of PA as a lead metabolite for RA-related inflammation but also reinforces the therapeutic rationale of targeting the NF-κB pathway in RA treatment. Notably, PA exhibited a relatively weak regulatory effect on MMP-3 mRNA expression, unlike its significant suppression of IL-6 and TNF-α. This suggests that the anti-inflammatory action of PA in RA-HFLS may be more specific to inhibiting the production of pro-inflammatory cytokines, with limited direct impact on mediators of joint matrix degradation, or may involve distinct mechanisms. Furthermore, the observed trend of slight MMP-3 upregulation at lower PA concentrations warrants attention, indicating a potential concentration- or pathway-dependent bidirectional regulatory effect. This finding provides a new direction for elucidating the precise regulatory mechanisms of PA.

From the perspective of medicinal potential, the therapeutic value of botanical drugs and their bioactive metabolites must be assessed by integrating both efficacy and safety considerations. Current first-line RA treatments, including MTX and biologics, are associated with well-documented adverse effects, underscoring the need for alternative therapeutic options with favorable safety profiles. PA is a pentacyclic triterpenoid widely distributed in plants used in traditional medicine [29,30]. In the present study, ADMET predictions indicated that PA possesses favorable intestinal absorption, low risk of drug–drug interactions, and good genetic safety, indicating favorable druggability. Regarding cytotoxicity, previous toxicological studies have reported that PA exhibits low cytotoxicity in normal cell lines, with PA showing an IC50 of 80 μM in normal BEAS-2B cells (human normal lung epithelial cells) [31]; in the present study, PA showed no significant cytotoxicity in RA-HFLS cells at concentrations ≤7 μM, consistent with these reports. However, ADMET predictions also indicated potential risks, including hepatotoxicity and reproductive toxicity. Additionally, pentacyclic triterpenoids, including PA, have been reported to modulate cytochrome P450 enzymes in vitro [32], suggesting potential interactions with conventional RA drugs metabolized through these pathways. Therefore, systematic safety evaluations—including chronic toxicity, reproductive toxicity, and drug interaction studies—are necessary before clinical application. Regarding bioavailability, ADMET predictions indicated that PA exhibits high lipophilicity (LogP = 5.15) but poor water solubility, consistent with reports that pentacyclic triterpenoids have limited oral bioavailability [30]. Pharmacokinetic studies in rats have shown that after oral administration, plasma concentrations of parent PA are low, but various active metabolites have been detected [33]. Collectively, although ADMET predictions and in vitro experiments support the druggability potential of PA, its in vivo efficacy and safety require further validation through integrated pharmacokinetic-pharmacodynamic studies and systematic toxicological investigations in animal models. Comprehensive toxicological characterization—including genotoxicity, subchronic toxicity, and reproductive toxicity studies—is beyond the scope of the current work but represents an important direction for future research prior to any potential clinical application of PA.

The network analysis approach employed in this study is inherently a prediction tool based on existing knowledge and structural similarity, and it possesses inherent limitations. Databases such as SwissTargetPrediction operate on the principle of “similar structure implies similar function,” which may lead to overestimation of certain target classes while potentially missing structurally novel but active targets. Furthermore, network analysis cannot account for the effective concentration of metabolites at target sites, metabolic processes, or cell type specificity. As highlighted in recent critiques of network-based approaches in pharmacology, these limitations manifest as a systemic bias: network analyses across diverse natural products and diseases consistently converge on a narrow set of molecules (e.g., quercetin, AKT1) and canonical pathways, suggesting that the outputs are driven more by database-dependent prediction tools than by genuine pharmacological specificity [34]. Many databases, including SwissTargetPrediction, rely on the “similarity ensemble approach,” which predicts targets based on structural similarity to known ligands. While useful for hypothesis generation, this approach is prone to overprediction and may generate false positives, particularly for structurally promiscuous metabolites. Conversely, genuinely novel targets with no structural precedent in the training data may be missed, leading to false negatives. Furthermore, these databases are often interconnected and not regularly updated (e.g., SwissADME has not been updated since 2019), which can perpetuate outdated or poorly curated information. Consequently, the network analysis presented here should be interpreted with appropriate caution; it serves solely to generate testable hypotheses and does not, on its own, constitute evidence of pharmacological activity. Similarly, while molecular docking can predict binding affinity, it cannot reflect the true binding dynamics within the complex intracellular environment. ADMET predictions are based on existing databases and algorithmic models, and carry the risk of both false-positive and false-negative results, particularly for toxicity predictions. All predictive findings require rigorous experimental validation, as we have initiated with the NF-κB pathway in the present study, and all other predicted targets and pathways remain to be confirmed in future investigations.

Importantly, our network analysis was based exclusively on the chemical structure of PA and its computationally predicted protein targets, rather than on any cell type-specific transcriptomic datasets. Nevertheless, the broader concern regarding cell type specificity remains valid. The predicted targets of PA are expressed across multiple cell types involved in RA pathogenesis, including fibroblast-like synoviocytes, macrophages, T cells, and neutrophils. However, the present study only validated the anti-inflammatory effects of PA in a single cell type—RA-HFLS. This represents a significant limitation of this in vitro study, as RA pathogenesis involves complex interactions between multiple cell types within the synovial microenvironment in vivo. Therefore, the observed inhibition of NF-κB signaling and inflammatory cytokine production in RA-HFLS represents only one component of PA’s potential anti-inflammatory mechanism. Future studies should investigate the effects of PA on other RA-relevant cell types, including macrophages, T cells, and osteoclasts, and employ co-culture systems or in vivo animal models of arthritis to fully elucidate the multi-cellular mechanisms underlying the therapeutic potential of PA in RA.

From an ethnopharmacological perspective, MDA has been used by the Dong ethnic group for its anti-inflammatory and analgesic properties in folk practice. The present study provides modern scientific evidence supporting this traditional use, demonstrating that PA exerts anti-inflammatory effects in an RA synovial cell model through NF-κB signaling pathway inhibition. These findings align with the principles of ethnopharmacological research, which seeks to validate traditional medical knowledge using contemporary scientific methods while respecting the cultural context of traditional medicine use. In conclusion, this study demonstrates that PA inhibits TNF-α-induced inflammatory responses in RA-HFLS cells by suppressing NF-κB signaling pathway activation, providing a scientific basis for the traditional use of MDA in inflammatory conditions and identifying PA as a potential lead metabolite for anti- inflammatory drug development. However, due to the inherent limitations of computational prediction methods and the use of a single cell type, further studies are warranted to investigate whether the anti-inflammatory effects of PA involve other signaling pathways or molecular targets, and to fully elucidate its therapeutic potential using animal models of arthritis and multiple RA-associated cell types.

## 4. Materials and Methods

### 4.1. Solution Preparation and Content Determination

Standard Solution Preparation: An appropriate amount of PA standard (purity ≥ 98%, Yuanye Bio, B25536, Shanghai, China) was accurately weighed, dissolved in 95% ethanol, and diluted to volume in a 10 mL volumetric flask to prepare a stock solution. Serial dilutions were prepared to obtain standard solutions with concentrations ranging from 0.00414 to 2.12 mg/mL. Solutions were filtered through a 0.45 μm microporous membrane and stored at 4 °C for immediate use to ensure standard activity.

Sample Solution Preparation: The plant material used in this study consisted of dried roots of *Potentilla freyniana* Bornm. (known as Madeng’ai, MDA). Three batches were collected: one from Youzha Street, Guizhou Province, China, in 2019, and two from Tongdao County, Hunan Province, China, in 2023 and 2024, respectively. The botanical identity was confirmed by Prof. Zaiqi Zhang (Hunan University of Medicine) and the plant material was provided by Dr. Liang Cao (Hunan University of Medicine). A voucher specimen (code: 201910PF) has been deposited at the International Laboratory for TCM and Ethnomedicine Innovation and Development, School of Pharmacy, Hunan University of Chinese Medicine (Changsha, China). No new genetic sequences were generated for this plant material in this study. The samples were pulverized, sieved through a 60-mesh sieve, and precisely 1.0000 g of each powder was weighed into a 25 mL stoppered conical flask, followed by the addition of 10 mL of 95% ethanol. After sealing, ultrasonic extraction was performed for 30 min (200 W, 40 kHz). The extract was then cooled to room temperature, centrifuged at 8000× *g* rpm for 10 min, and the supernatant was filtered through a 0.45 μm membrane. The filtrate was collected as the sample solution and stored at 4 °C until analysis.

Chromatographic Conditions: Analysis was performed using an Agilent Poroshell 120 EC-C18 column (4.6 × 150 mm, 4 μm). The column temperature was maintained at 25 °C. The detection wavelength was set at 205 nm. The mobile phase was 0.1% formic acid in water (Phase A) and methanol (Phase B,) in a volume ratio of 20:80 under isocratic elution. The flow rate was 1.0 mL/min, injection volume was 10 μL, and the analysis time per sample was 15 min.

Method Validation and Content Calculation: Standard solutions were injected under the above conditions, and peak areas were recorded. A standard curve was plotted with PA concentration (X, mg/mL) as the abscissa and peak area (Y) as the ordinate to obtain the regression equation and correlation coefficient (R^2^). Sample solutions were injected, and PA content (mg/g) in each batch was calculated using the regression equation.

### 4.2. Network Analysis for Predicting Potential Targets and Pathways of PA Against RA

Target Prediction: As a hypothesis-generating approach, potential targets of PA were predicted using the TCMSP database (https://www.tcmsp-e.com/, accessed on 1 November 2025) and SwissTargetPrediction platform (http://www.swisstargetprediction.ch/, accessed on 1 November 2025), with the understanding that all predictions require subsequent experimental validation.

Disease Target Retrieval: RA-related disease targets were retrieved from GeneCards (https://www.genecards.org/, accessed on 1 November 2025) using the keyword “rheumatoid arthritis”. To ensure data quality and relevance, only genes with a relevance score ≥ 5 were selected. Additional RA-related targets were obtained from the OMIM database (https://www.omim.org/, accessed on 1 November 2025) using the same keyword, including all manually curated genes with documented associations to RA.

Intersection Target Identification: After merging targets from both databases and removing duplicate entries using the Venny 2.1 online tool (https://bioinfogp.cnb.csic.es/tools/venny/, accessed on 15 January 2026), the intersecting targets between PA-predicted and RA-related genes were identified.

PPI Network Construction: These intersecting targets were imported into the STRING database (https://string-db.org/, accessed on 15 January 2026) to construct a protein–protein interaction (PPI) network.

Functional Enrichment Analysis: Functional annotation and pathway enrichment analysis of the intersecting targets were performed using the DAVID database (https://davidbioinformatics.nih.gov/, accessed on 15 January 2026), with parameters set to official gene symbols and species limited to Homo sapiens. Gene Ontology (GO) enrichment analysis, covering Biological Process, Cellular Component, and Molecular Function, and Kyoto Encyclopedia of Genes and Genomes (KEGG) pathway enrichment analysis were conducted. Enrichment significance was set at a false discovery rate (FDR)-corrected *p* < 0.05. Core GO terms and KEGG pathways were visualized based on enrichment significance (−log10 *p* value).

### 4.3. Molecular Docking Analysis

The 2D structure of PA was obtained from the PubChem database (http://pubchem.ncbi.nlm.nih.gov/, accessed on 20 February 2026) and converted to 3D format using ChemOffice software, then saved as a mol2 file. Protein targets were selected from the RCSB PDB database (http://www.rcsb.org/, accessed on 20 February 2026), and crystal structures with high resolution were chosen as receptors for molecular docking. Preprocessing steps, including removal of water molecules and phosphate groups, were performed using PyMOL 2.6, and the structures were saved in PDB format. AutoDock 1.5.6 was used to add hydrogen atoms and assign torsion angles to both the protein and ligand, followed by determination of docking grid coordinates. Molecular docking was conducted using AutoDock Vina, and the optimal binding conformations were selected based on binding energy scores. Finally, docking results were visualized using Discovery Studio 2019 and PyMOL 2.6 to generate 2D interaction diagrams and 3D binding mode analyses.

### 4.4. ADMET Prediction Analysis

ADMET prediction was performed using three online platforms: SwissADME, admetSAR 3.0, and ProTox-3.0, to evaluate the physicochemical properties, pharmacokinetics, drug-likeness, and potential toxicity of PA. Key parameters assessed included water solubility, lipophilicity (LogP), gastrointestinal absorption, blood–brain barrier penetration, P-glycoprotein substrate status, cytochrome P450 enzyme inhibition, and compliance with drug-likeness rules. Toxicity endpoints included acute oral toxicity, hepatotoxicity, respiratory toxicity, reproductive toxicity, repeated-dose toxicity, Ames mutagenicity, skin sensitization, and hERG cardiotoxicity. Results from the three platforms were cross-validated to ensure reliability of the predictions.

### 4.5. Cell Culture and Inflammatory Model Establishment

RA-HFLS cells (Otwo Biotech, HTX1835, Shenzhen, China) were thawed rapidly in a 37 °C water bath, transferred to a centrifuge tube with 5 mL of RA-HFLS-specific medium (Otwo Biotech, HTX1835M, Wuhan, China), and centrifuged at 1000 rpm for 5 min. The supernatant was discarded, and cells were resuspended in fresh complete medium, transferred to a culture flask, and maintained at 37 °C with 5% CO_2_ in a humidified incubator (Xiangyi, L535R, Changsha, China). Medium was changed every 24–48 h. Cells at 80–90% confluence were passaged using 0.25% trypsin. Cells from passages 3–5 in good condition and in the logarithmic growth phase were used for experiments.

Screening of Optimal TNF-α Induction Concentration: RA-HFLS cells were seeded in a 96-well plate at 5 × 10^3^ cells/well with 100 μL complete medium. After 24 h for adherence, medium was replaced with fresh medium containing different concentrations of TNF-α (0, 10, 20, 30, 40, 50 ng/mL) (Peprotech, 300-01A, Cranbury, NJ, USA). Each group had three replicates. After 24 h incubation, cell viability was assessed by CCK-8 assay. Cell viability (%) = [(OD_sample − OD_blank)/(OD_control − OD_blank)] × 100%. The TNF-α concentration inducing significant cell proliferation was selected as the optimal concentration.

### 4.6. Drug Preparation and Cytotoxicity Assay

Drug Preparation: The PA (purity ≥ 98%, Aladdin, P418619, Shanghai, China) used in all cell-based experiments was a commercially purchased standard. PA standard and methotrexate (MTX) (labeled content: 2.5 mg/tablet, Shanghai Sine Pharmaceutical, 107240102, Shanghai, China) were dissolved in DMSO (Sigma, D4540, St. Louis, MO, USA) and PBS, respectively, to prepare stock solutions. Working concentrations were prepared by dilution with cell-specific medium, ensuring final concentrations of DMSO and PBS were below 0.1% in all groups. Corresponding solvent control groups were included.

PA Cytotoxicity Assay: RA-HFLS cells were seeded in a 96-well plate at 5 × 10^3^ cells/well. After 24 h of adherence, medium was replaced with fresh medium containing different concentrations of PA (0, 1.75, 3.5, 7, 14, 28 μM). Each treatment group was set up in triplicate. After 24 h incubation, cell viability was measured by CCK-8 assay: medium was carefully removed, 100 μL of serum-free medium containing 10% (*v*/*v*) CCK-8 solution (GLPBIO, GK10001, Montclair, CA, USA) was added per well, and plates were incubated for 1.5 h. Absorbance (OD) at 450 nm was measured using a microplate reader (Berthold Technologies Bioanalytic, Singapore). Cell viability (%) = [(OD_sample − OD_blank)/(OD_control − OD_blank)] × 100%. To ensure that the observed effects in subsequent experiments were not due to basal cytotoxicity, PA concentrations resulting in a cell viability > 90% were selected as the low, medium, and high concentrations for the following studies. MTX Cytotoxicity Assay: The procedure was identical to the PA cytotoxicity assay, with PA replaced by different concentrations of MTX (0, 0.1, 0.5, 1, 5, 10 μM). After 24 h, cell viability was assessed. The MTX concentration yielding a cell viability >90% was selected as the positive control concentration.

### 4.7. Experimental Grouping and Intervention

Based on the above results, six groups were established: (1) Control group: RA-HFLS specific medium only. (2) TNF-α model group: Medium containing 50 ng/mL TNF-α. (3) TNF-α + PA low concentration group (PA-L): Medium containing 50 ng/mL TNF-α + 1.75 μM PA. (4) TNF-α + PA medium concentration group (PA-M): Medium containing 50 ng/mL TNF-α + 3.5 μM PA. (5) TNF-α + PA high concentration group (PA-H): Medium containing 50 ng/mL TNF-α + 7 μM PA. (6) TNF-α + MTX group (MTX): Medium containing 50 ng/mL TNF-α + 0.5 μM MTX (positive control).

Cell Seeding and Intervention: RA-HFLS cells were seeded at appropriate densities in different culture plates according to the requirements of the specific assays. For the CCK-8 assay, cells were seeded in 96-well plates at a density of 5 × 10^3^ cells per well. For the ELISA assay, cells were seeded in 96-well plates at a density of 1.5 × 10^4^ cells per well. For WB and RT-qPCR analyses, cells were seeded in 6-well plates at a density of 5 × 10^4^ cells per well. After seeding, all plates were placed in an incubator for culture. For the CCK-8 and ELISA experiments, interventions were initiated after 24 h of incubation to allow for cell adhesion. For the WB and RT-qPCR experiments, interventions were applied when the cells reached 80–90% confluence. Based on reports that pre-treatment with natural metabolites can enhance anti-inflammatory effects, cells were pre-treated for 2 h with media containing the specified concentrations of PA or MTX. Subsequently, TNF-α was added (final concentration: 50 ng/mL) for co-stimulation. The duration of co-stimulation varied by assay: 24 h for CCK-8 and ELISA; 1 h for RT-qPCR and WB.

### 4.8. CCK-8 Assay for Cell Proliferation

Following the 24 h intervention, the CCK-8 assay was performed. The culture medium was carefully aspirated from each well to avoid disturbing the adherent cells. Subsequently, 100 µL of serum-free medium containing 10% (*v*/*v*) CCK-8 solution was added to each well. The 96-well plate was gently shaken to ensure even distribution of the solution and then incubated for 1.5 h at 37 °C in a 5% CO_2_ incubator, protected from light. After incubation, the absorbance at 450 nm was measured using a microplate reader. Wells containing only 100 µL of the serum-free medium with 10% CCK-8 solution (without cells) served as the blank control. Each experimental group was set up in triplicate. The average optical density (OD) value for each group was calculated, and cell viability was determined to analyze the effect of PA on TNF-α-induced RA-HFLS cell proliferation.

### 4.9. ELISA for Inflammatory Cytokine Secretion

After the 24 h treatment, cell culture supernatants were collected. The supernatants were centrifuged at 12,000× *g* for 10 min at 4 °C to remove cellular debris, then aliquoted and stored at −80 °C until analysis. The experiments were strictly performed according to the manufacturer’s instructions for the human TNF-α ELISA kit (Solarbio, SEKH-0047, Beijing, China) and the human IL-6 ELISA kit (NeoBioscience, EHC007, Shenzhen, China). Briefly, after all reagents and samples were equilibrated to room temperature, standards or samples (100 µL per well) were added to the appropriate wells of the pre-coated plate and incubated at 37 °C for 2 h. After incubation, the plate was washed three times with wash buffer and thoroughly blotted dry. Then, 100 µL of detection antibody working solution was added to each well and incubated at 37 °C for 1 h, followed by another washing cycle. Next, 100 µL of enzyme conjugate working solution was added per well and incubated at 37 °C for 30 min, after which the plate was washed again. Subsequently, 100 µL of substrate solution was added to each well, and the plate was incubated in the dark at 37 °C for 15 min. Finally, 50 µL of stop solution was added to each well to terminate the reaction. The absorbance at 450 nm was immediately measured using a microplate reader. Standard curves were generated by plotting the known concentrations of the standards against their corresponding OD values, which were then used to calculate the concentrations of TNF-α and IL-6 in the samples.

### 4.10. Western Blot for Protein Expression

Total protein was extracted using RIPA lysis buffer with protease and phosphatase inhibitors. Protein concentration was determined by BCA assay (SEVEN, SW101-02, Beijing, China). Equal protein amounts (30 µg) were denatured, separated by 10% SDS-PAGE, and transferred to PVDF membranes (Merck, IPVH00010, Darmstadt, Germany). Membranes were blocked and incubated overnight at 4 °C with specific primary antibodies: p-p65 (65kDa, CST, 3033T, Danvers, MA, USA), p65 (65kDa, ABclonal, Wuhan, A19653, China), p-IκBα (39kDa, Affinity Biosciences, AF2002, Changzhou, China), IκBα (39kDa, Abclonal, A19714, Wuhan, China), and GAPDH (37kDa, Abbkine, A01020-SR, Beijing, China) as loading control. After washing, membranes were incubated with HRP-conjugated secondary antibody for 1 h at room temperature, followed by ECL detection. Band intensity was analyzed using Image J software (version 1.53a, National Institutes of Health, USA). Ratios of p-IκBα/IκBα, IκBα/GAPDH and p-p65/p65 were calculated.

### 4.11. RT-qPCR for Inflammatory Cytokine mRNA Expression

Total RNA was extracted using TRIzol, and concentration/purity (A260/A280 1.8-2.0) was determined. Equal RNA amounts (up to 500 ng) were reverse transcribed to cDNA using PrimeScript™ RT Master Mix (TaKaRa, RR036A, Kusatsu, Shiga, Japan). Quantitative PCR was performed using TB Green Premix Ex Taq™ II (TaKaRa, RR820A, Kusatsu, Shiga, Japan) on a LightCycler 480 system to detect mRNA expression of IL-6, IL-1β, TNF-α, and MMP-3. GAPDH served as the internal reference. Primer sequences are listed in Table 4. Each sample was run in triplicate. Relative expression was calculated using the 2^−ΔΔCt^ method.

### 4.12. Statistical Analysis

All experiments were independently repeated at least three times. Statistical analysis was performed using GraphPad Prism 10.1.2. Comparisons among multiple groups were analyzed by one-way ANOVA followed by Bonferroni’s post hoc test for multiple comparisons. Data are presented as mean ± standard error of the mean (SEM), with *p* < 0.05 considered statistically significant.

## 5. Conclusions

The present study demonstrates that PA, a bioactive metabolite of the Dong botanical drug MDA, suppresses the expression and secretion of inflammatory cytokines in TNF-α-induced RA-HFLS cells by inhibiting NF-κB signaling pathway activation, thereby exhibiting potential anti-inflammatory activity in RA-HFLS. Network pharmacology, molecular docking, and ADMET predictions further support the binding affinity of PA to core targets of the NF-κB pathway and its favorable druggability, providing preliminary evidence for its further development. However, the present study has limitations, including the inherent constraints of computational prediction methods, the validation of only the NF-κB pathway among multiple predicted targets (e.g., TLR4 and JAK2), the use of a single cell type, and the lack of in vivo data. Further validation in animal models and multiple RA-associated cell types is warranted.

## Figures and Tables

**Table 1:** Determination of PA content in different batches of MDA samples (*n* = 3).

Batch (Origin, Year)	Weight (g)	Peak Area (mAU·s)	Content (mg/g)	Mean Content (mg/g)
CL-17-1-1 (Guizhou, 2019)	1.0035	82.62394	0.1077	0.1117
	1.0025	85.20592	0.1131	
	1.0089	86.16497	0.1144	
CL-17-1-7 (Huaihua, 2023)	1.0015	56.42893	0.0538	0.0504
	1.0004	55.09828	0.0511	
	1.0005	52.74940	0.0463	
CL-17-1-8 (Huaihua, 2024)	1.0011	40.38166	0.0208	0.0237
	1.0006	42.01608	0.0242	
	1.0007	43.03247	0.0262	

**Table 2:** Molecular docking binding energies of PA with NF-κB pathway targets.

Target Protein	PBD ID	Binding Energy (kcal/mol)
IKKβ	4KIK	−8.8
P65	1NFI	−7.8
IκBα	1IKN	−7.9

Note: Binding energy ≤ −5.0 kcal/mol indicates a strong binding potential between the ligand and the receptor.

**Table 3:** ADMET prediction for PA.

Category	Index	Prediction
Physicochemical	Molecular weight	472.71 g/mol
	TPSA	77.76 Å^2^
	Consensus LogP	5.15
	Water solubility	1.7 × 10^−4^ mg/mL (poor)
Pharmacokinetics	Gastrointestinal absorption	High
	BBB permeability	Negative
	P-gp substrate	Yes
	CYP450 inhibition	No inhibition
Drug-likeness	Lipinski rule	1 violation
	Veber rule	Compliant
	Bioavailability score	0.56
	PAINS alert	No
Toxicity	Ames test	Negative
	Skin sensitization	Negative
	hERG toxicity	Negative
	Acute oral toxicity	Positive
	Hepatotoxicity	Positive
	Respiratory toxicity	Positive
	Reproductive toxicity	Positive
	Repeated dose toxicity	Positive

Note: Absorption, distribution, metabolism, excretion, and toxicity (ADMET) prediction was performed using three online platforms: SwissADME, admetSAR 3.0, and ProTox-3.0. A molecular weight < 500 and TPSA < 140 Å^2^ meet the oral drug requirements; LogP > 5 indicates high lipophilicity; a bioavailability score ≥ 0.55 suggests favorable drug-likeness.

**Table 4:** Primer sequences for qPCR.

Gene	Forward Primer (5′ to 3′)	Reverse Primer (5′ to 3′)
IL-6	CCCTGACCCAACCACAAA	GGACTGCAGGAACTCCTTAAA
TNF-α	CCTCTCTCTAATCAGCCCTCTG	GAGGACCTGGGAGTAGATGAG
IL-1β	CAGGCTGCTCTGGGATTCTC	GTCCTGGAAGGAGCACTTCAT
MMP-3	TGGACAAAGGATACAACAGGGAC	ATCTTGAGACAGGCGGAACC
GAPDH	TCGGAGTCAACGGATTTGGT	TTCCCGTTCTCAGCCTTGAC

## Data Availability

The raw data supporting the findings of this study are included in the article. Further inquiries can be directed to the corresponding author.
